# Miscellanies

**Published:** 1841-04-01

**Authors:** 


### Miscellanies.

### Medical Reform.

At the time we are writing (the latter end of March) it is difficult to say what aspect this wears. Mr. Warburton's Bill has shuffled off its mortal coil, one scarce knows how or where, although it is obvious *why*. And the imprecations that have been heaped upon it must be highly satisfactory to the honourable member for Bridgewater, and put him much in love with the cause of Medical Reform.

Mr. Hawes's Bill has been treated scurvily too. The "ridiculus mus" has crept out of it to the delight of some and the dismay of many. Whether the honourable member for Lambeth may be encouraged to go on, we cannot venture to determine.

In the mean time, there are two significant indications of what turn matters are like to take. *First,* there are such differences of opinion among reformers, that unanimity in recommending or in prosecuting any scheme, seems far off and unapproachable; and *secondly,* the Colleges have admitted more or less the necessity for modifications in their constitution. The conclusions which may be naturally drawn from these premises appear to us,—the improbability of any strong interference on the part of the legislature—and the probability of spontaneous changes, more or less satisfactory and considerable, on the part of the corporations. As this will, in all likelihood, be the denouement of the piece, we are the more inclined to gather and to dwell upon the reform manifestoes of those bodies.

And first of the College of Physicians of Edinburgh. They have published the following Report.

At a meeting of the Royal College of Physicians of Edinburgh, called to consider the Report of a Committee of their body, on the Bills for Medical Reform, which have been introduced into the House of Commons by Mr. Warburton and Mr. Hawes, the following Resolutions were unanimously adopted:—

1st. That the College derive much pleasure from finding that the subject of Medical Reform, which has so often been under their consideration, and in which they have repeatedly endeavoured to interest the legislature, has at last been brought, in a tangible form, under the notice of the House of Commons; and trust, that the full discussion which the subject has received, and is receiving, from the profession at large, and the attention now about to be bestowed on it by Parliament, will lead to the removal of some of the evils of which the College have frequently had occasion to complain.

2d. That, in the opinion of this College, the great evil arising from the want of an uniform system of medical legislation throughout the united kingdom, is the possession, by particular corporations, of local privileges, which render their Licentiates alone legally capable of acting as general practitioners in particular districts and portions of the country, to the exclusion of persons of equal, and it may even be, of superior qualifications.

3d. That, so far as the College is aware, the only plausible objection which has been urged against the abolition of these local privileges, and against the adoption of a system for placing the Licentiates of all the Medical Corporations on an equal footing in respect of the right of practice, is the inequality alleged to exist, or actually existing, in the amount of Medical Education required of candidates for their licenses by the several Boards, and in the degrees of strictness with which the examinations of such candidates are conducted.

4th. That whilst, therefore, with a view to the interests both of the public and the profession, community of privilege should, in the opinion of this College, be the primary object of any legislative enactment relative to the medical profession, sufficient education and examination must, at the same time, be duly provided for.

5th. That from the communications which have taken place, and the understanding that has been come to, between a number of the different Boards with which the superintendence of medical education at present rests, little difficulty can now exist in fixing a minimum course of study, general and professional, without evidence of having passed through which, no one should be allowed to present himself as a candidate for a medical license. On this point the College will only farther refer to the joint resolutions agreed on by the Medical Faculty of the University, and by the Royal Colleges of Physicians and Surgeons, of Edinburgh, of date October 1838.

6th. That to produce some approach to uniformity in the system on which the examinations of candidates for licenses are conducted by the several Boards, and to secure the public against the admission of incompetent persons into the medical profession, it appears to this College that it would be desirable that some superintending body should be constituted, having authority to take cognizance of the manner in which the duty of examination is executed.

7th. That the persons of whom this Board should consist might probably be most advantageously selected by the Crown from lists furnished by this and the other boards at present entrusted with the government of the medical profession.

8th. That, in the opinion of this College, no measure of medical reform will be satisfactory which does not confer on a person who has once received a certicate of his fitness to exercise the medical profession from any of the established Boards, the right of practising in any district of the country, or in any particular department of the profession, without the necessity of submitting himself to a second examination before another board.

9th. That whilst the College readily acknowledge that the proposal of creating a representative body or bodies, elected periodically by the profession at large, by which the affairs of the medical profession might be superintended and directed—(a proposal which forms so prominent a feature in the two measures which have been submitted to Parliament)—is desired by many most respectable members of the profession, they are disposed to believe that this desire has in a great measure originated in accidental and removable causes; and they are satisfied that any attempt to carry it into effect would be attended with serious inconveniences, if not insuperable difficulties.

10th. That, in particular, the College conceive that this proposal has in a great measure grown out of the dissatisfaction very generally prevailing among the members of the medical profession, not only with the local privileges of practice attached to the licentiates of particular corporations, as already noticed, but also with the narrow and exclusive system on which admission into the governing body of some of the medical incorporations has hitherto been regulated; and that the adoption by, or enforcement on, these corporations of conditions of admission of a more reasonable and liberal character, in obtaining for them the confidence of their licentiates, would in a great measure supersede the desire at present existing for a representative system of superintendence.

11th. That among the obvious inconveniences with which the election of a representative body, by the medical profession at large, would be attended, the College conceive that it would have the effect of producing and continually renewing agitation and dissension among the members of the profession, and of directing their attention from far more important duties; whilst those who are best qualified for performing the duties that should be committed to a superintending body, would be least likely and least able to take those steps which are essential to the gaining of popular suffrages. And, as a farther objection to the Boards proposed, in the bills before Parliament, to be established for the regulation of the profession, it may be remarked, that, from the multifarious duties intended to be committed to their members, and the necessity which would be imposed upon them from time to time, of leaving their homes for execution of these duties, it would be impossible to obtain the services of persons of eminence and station in the profession; and that the appointments would therefore fall into the hands of an inferior grade of individuals, in whom neither the public nor the profession would have confidence.

12th. That whilst the College readily acknowledge that the actual constitution of some of the medical corporations in the united kingdom requires to be rendered more conformable to the spirit of the age, they are not disposed to admit that these institutions are so useless, or so incapable of amendment, as to make it advisable either to abolish them by direct, or to supersede them by indirect legislation, the acknowledged fact being that, notwithstanding any defects under which they may labour, the country is, through their agency, provided, at the present time, far beyond all precedent, with well-educated and judicious practitioners.

13th. That it would be desirable that the duty of examination should be remunerated in such a manner as to remove the possibility of a suspicion, that the examiners have a pecuniary interest in the number on whom they confer the license to practise.

14th. That, considering the large amount of services gratuitously rendered to the public by the medical profession, the very inadequate compensation received by a large proportion of its members for the long and expensive course of education necessary to qualify them to practise, and for the performance of its very laborious duties; and considering also the direct interest which the public has in being able easily to distinguish between qualified and unqualified practitioners of the healing art, the College are decidedly of opinion, that any additional expenses which might be occasioned by an improved system of medical legislation should be defrayed out of the public purse, and not by an annual tax upon the profession, as seems to be contemplated in the measures which have been submitted to Parliament.

15th. That the College consider that it would be highly desirable that a scheme should be arranged for the registration of licensed practitioners. That, in their opinion, none but registered practitioners should be legally eligible for any public professional situation; that the assumption of a professional designation by a person not entitled to it, should be declared an offence punishable at common law; and that the right of suing in Courts of law for professional remuneration should be secured to licensed, and denied to unlicensed practitioners. But, in recommending enactments to these effects, for the encouragement of the licensed, and the discouragement of the unlicensed, the College is convinced that the suppression of unlicensed practitioners is beyond the reach of legislative interference, so long as there exists a taste for this species of practice in the public mind.

Such reform would be a very good thing for the Graduates of Edinburgh. For it is *not very* likely that many Englishmen will either go to Scotland for practice, or, if they go, do any thing but starve there—while it *is very* likely that Scotchmen will come to England and do well here. So that the reform which the College of Physicians of Edinburgh desire would be a capital hit. Universal legislation of practice would tell bravely for the modern Athens. A cheap education would be got there, and its schools would thrive.

We now come to the—

### College of Physicians of London.

*Report addressed to the Royal College of Physicians by the Committee appointed by the College to confer with the Deputation from the College of Surgeons and the Society of Apothecaries.*

" The Committee appointed by the College of Physicians to confer with the deputation from the College of Surgeons, and the Society of Apothecaries, having carefully investigated the various grievances complained of in the several petitions to parliament for Medical Reform, and having considered the communications from different fellows 'of their views, as to any or what changes in the present constitution of the college can be effected with safety to the great objects of the college,' submits the following report:—

The grievances alleged in the petitions for reform may be stated as follows:—

1. The want of a general registration of licensed practitioners.

2. The absence of uniformity of education and qualifications in England, Ireland, and Scotland, and that licenses to practise obtained in one country are invalid in the others.

3. Self-election to the fellowship of the College of Physicians, to the council of the College of Surgeons, and to the court of assistants and examiners of the Society of Apothecaries.

4. The exclusion of the licentiates of the College of Physicians from the use of the library and museum of the college.

5. The want of some legislative enactment respecting the licensing of duly qualified persons as chemists and druggists.

6. The want of some body or board to whom all questions of medical police, public health, &c., should be referred.

7. The absence of some restriction upon quacks and vendors of quack medicines.

With respect to the last complaint, the necessity for some restrictions upon quacks or quack medicines, the committee is of opinion, that any future legislative enactments upon that subject, if such were deemed advisable, should be entirely irrespective of the College of Physicians, and should demand only the interference of the civil magistrate.

As regards the other allegations contained in the petitions, the committee think that certain changes may be effected with safety, and, in its judgment, with advantage to the College of Physicians; and which will remedy the evils complained of, as far as they relate to the college.

The committee, fully appreciating the difficulty of the task confided to it, begs to submit to the college the following statement of the alterations which it believes to be desirable at the present time.

### *Resolutions of the Committee.*

1. That it is expedient that all physicians now practising throughout England and Wales, with a diploma of M.D. obtained from any British university, and who have attained the age of twenty-six years, should be entitled to admission into the order of licentiates of the college, without any examination, but upon the payment of fees hereafter to be determined.

2. In order to do away with the principle of self-election, the licentiates of the college shall henceforth nominate, annually, a certain number from their own body, for election into the fellowship, and from whom the fellows shall select one-half. The nomination to be conducted by ballot, a balloting-paper having been transmitted, on a given day, to each licentiate, whose address appears on the college list. The number of licentiates to be nominated in each year to be determined by the college.

3. That henceforth the licentiates shall, under certain regulations, have access to the library and museum of the college.

4. That the University of London having required for the degree of M.D. a high standard of education, which is, to a great extent, in accordance with the views of the College of Physicians, the college will be ready to admit into the order of licentiates the doctors in medicine of that university, provided that they shall respectively have attained the age of twenty-six years, and that the censors shall have assisted at their medical examinations.

5. That similar or equivalent privileges shall be accorded to the graduates in medicine of Oxford and Cambridge, who have obtained their license to practise, provided those universities shall have adopted a curriculum of medical study equal to that which the college requires.

6. That it is desirable that uniform medical qualifications should be demanded of all candidates for the degree of M.D. in England, Ireland, and Scotland, and that the degree of M.D. so obtained in either country should henceforth confer a right to practise in all, provided the graduate shall have enrolled himself in the College of Physicians of the country where he resides.

7. That doctors in medicine from foreign universities, or from such British universities as shall not assimilate their qualifications for the degree of M.D. to those contemplated in the foregoing resolution, shall be admitted into the order of licentiates upon producing testimonials of having fulfilled the course of medical study now enjoined by the college, and after having undergone the usual examinations by the censors.

8. That the College of Physicians should have only one board of examiners and a uniform system of examination for all candidates for their license, and that the order of extra-licentiates should be abolished.

9. That in any new legislative enactments that might be necessary to carry the foregoing resolutions into effect, powers might be vested in visitors appointed by the crown (or in some other controlling body), to whom all new by-laws of the College of Physicians should be submitted for their approbation.

Lastly. The committee is of opinion that if the fellows of the College of Physicians should express their willingness to modify their statutes to meet the wishes of physicians throughout the country, and to facilitate the admission into their body of all duly-educated persons, by the altering the mode of election into the fellowship, they would be authorised to claim from the legislature a confirmation and extension of the jurisdiction of the college, so as to render it effective for the protection of the interests of their branch of the profession throughout England and Wales."

This plan is a continuation of the half-measures hitherto pursued, which have reduced the college to that state of bankruptcy which its friends deplore. The distinction of fellows and licentiates is to be perpetuated—the latter are still to be elected by the former, made fellows by favouritism, excluded by jealousy. The amalgamation of physicians in England and Wales will be as remote as before. Whatever may be thought of one FACULTY, there can be little doubt of the absurdity of two orders of physicians—at all events of two orders not based on examinations open to all, but on election and intrigue.

### The Apothecaries' Report.

In conformity with the second resolution passed at the Conference held at the Royal College of Physicians, on the 21st of November, the Society of Apothecaries have taken into their consideration the acts of parliament granted to them in the years 1815 and 1835 (which last expired in the ensuing year), and have prepared a series of alterations and amendments in their act, which, if adopted, would in their opinion be highly acceptable to the general practitioner, and tend in a great measure to remove many objections that have from time to time been urged against that measure. It will be evident upon examination, that in the changes thus proposed by the Society they are not actuated by any interested motives, but that they have endeavoured to direct their attention solely to the correction of those imperfections which have been pointed out to them by their own experience, or by that of others.

But independent of the changes proposed in their act, the Society have thought it incumbent upon them, in accordance with the resolution above-mentioned, to offer a sketch of a more general and extensive measure, pointing out certain principles, which, if agreed to by the three existing corporations, would, they conceive, prove highly satisfactory to all parties, both in and out of the profession, and which they would be prepared to develop more at large if called upon so to do.

*The Amendments and Alterations proposed in the Act of* 1815.

1. The Society desire to give up the power of searching shops.

2. They wish to modify the clause compelling an apprenticeship of five years to an apothecary; they would propose either to shorten the period of apprenticeship, or receive as an equivalent a certain period of instruction (two years) in practical pharmacy, at the option of the parent or guardian of the pupil.

3. They would give up the power of prosecuting the unqualified practitioner, as they think it objectionable as now conducted. They think that the punishment of the unqualified practitioner should ensue upon summary conviction; and to render this effectual, it would be necessary.

4. To introduce a general registration of all medical men.

5. They would consent to the election of a certain proportion of the members of the Court of Examiners (not exceeding one-half) from among their licentiates of ten years' standing, not being members of the Society of Apothecaries; the election, however, still remaining as at present with the Society.

6. They would wish that one uniform sum should be paid for the certificate of qualification, both for London and the country; that sum to be 6*l.* 6*s.*

*Additions required to be made to the Act.*

1. Apprentices to Surgeons should be admitted to examination.

2. Army and Navy Surgeons, and Assistant-Surgeons, as well as those in the service of the East India Company, should be permitted to practise without being subjected to any further examination.

3. A clause should be inserted, compelling all chemists and druggists to undergo an examination in the Latin Pharmacopœia, Pharmaceutical Chemistry, and the Materia Medica, and granting them thereupon a license to carry on their business as chemist and druggist merely.

4. A clause providing for the examination of all persons practising midwifery.

Should such an arrangement of the act of parliament meet the wishes of the Royal Colleges of Physicians and Surgeons, it would be necessary that the latter body and the Society of Apothecaries should cordially unite in forming a curriculum of study that would apply equally to the surgical and medical student, and that they should also agree to a division of the subjects of examination, the College examining in Anatomy, Physiology, Surgery, and perhaps Midwifery; the Hall in Latin, Botany, Chemistry, Materia Medica, Forensic Medicine, and the practice of Medicine.

It would also be very desirable to divide the examination into two or more parts, to be held on separate days, or after certain intervals of time interposed between each.

*Enumeration of certain grounds upon which a more general and extensive measure might be founded.*

It is considered that no plan can be satisfactory to the general mass of the medical profession that does not concede some share in the management of each body to its respective members.

2. It is no less desirable that a uniformity of education, and also of examination, should be established in all the medical bodies in the three kingdoms, and that no impediment should exist to prevent the licensed practitioner from practising in whatever part of the empire he chose.

### Obituary.

Death has proved himself a dexterous marksman, of late, among our metropolitan Surgeons. Four prominent characters have, within a few months, been "gathered to their forefathers!" Copland Hutchinson, Anthony Carlisle, John Howship, and last, not least, Astley Cooper, have "left the warm precincts of the cheerful day," and gone to their narrow, but everlasting beds! None of these have died of old age, and the death of most of them was little expected. It is worthy of notice that two out of the four (Hutchinson and Cooper) died of the same complaint—organic disease of the heart or its consequences. Sir Astley Cooper having been long at the head of the surgical profession in this country, his illness excited more anxiety, and his death produced a stronger sensation, both in and out of the medical world, than we ever before witnessed. We think it is indisputable that no surgeon in this, or any other country, ever realized such a fortune, or acquired such wide spread fame, as Sir Astley Cooper. Much, both of his riches and reputation, was owing to his pleasing manners and cheering conduct towards his patients, and his liberal and honorable bearing towards his professional brethren. The eccentricities and uncouthness of Abernethy threw a handsome fortune into the purse of his more polished and courtly contemporary.*

It was once the fashion among hypercritics to say that Sir Astley Cooper was not a very scientific Surgeon. But neither medicine nor surgery has yet attained the rank of science; and we are inclined to think that he who is the most successful is the most scientific practitioner. Besides, these detractions have been put out of countenance by the valuable surgical works, and the beautiful anatomical preparations bequeathed to the profession by Sir Astley Cooper.

Although we have been personally acquainted with this distinguished member of our profession for 36 years past, yet his character has been so public that we can say little that has not been already said of him. The readers of this Journal are aware of the high terms in which we have always spoken of this eminent surgeon and his works, and we will not now indulge in panegyric, merely because he is dead.

Sir Astley exhibited a remarkable illustration of the adage—that it is more difficult to know one's self than to know one's neighbour. His long practice in the city must have brought many proofs before him of the difficulty as well as danger of changing long-established habits, whether mental or corporeal. Yet before the grand climacteric had passed over his head, he determined to break from the turmoil of overwhelming practice, and return to the woods, the lawns, and the groves of his elegant Tusculum, near Watford—there to crown

"A youth of labour with an age of ease."

A very short trial of this "otium cum dignitate," convinced the worthy Baronet that the subduction of mental excitement and corporeal exertion, in his case, would be attended with the same consequences that result from the sudden withdrawal of opium or ardent spirits from those who long indulged in such stimuli. The struggle was short, for the *ennui* was insupportable; and Sir Astley put on the professional harness never to be thrown off, till death closed the scene! The bustle and activity of body, the energy of mind, and the daily or rather hourly incense, to which the late Baronet was so long accustomed, could not be exchanged for mere literary leisure or rural pursuits, and the blackest shade of melancholy, or perhaps something worse, would soon have wrapped a noble mind in impenetrable gloom, had there not happily remained an open door for re-entering the busy scene of professional life.

---

* The immediate cause of death in both cases, was the same—Effusion in the Chest.

The anecdotes of Sir Astley's life would make a small volume; for he had a great deal more of humour, especially of good humour, than his eccentric contemporary, Abernethy, and seldom lost an opportunity of exercising it himself, or extracting it from others. A good many of his *facetiæ* are recorded in his lectures, but the great bulk are only floating in the memory of his friends and acquaintances. The bon mots which he made or picked up, were almost innumerable. We shall only allude to one—which he was very fond of repeating. When he removed a small tumour from the head of the then Prince Regent, his royal patient asked him what the tumour was called? Sir A. replied that its technical name was a "STEATOME." "Indeed!" rejoined the Prince, "I hope sincerely that it will *stay-at-home* for the future, and pay me no more visits."

The personal appearance of the worthy Baronet was extremely propossessing, and, combined with his affability, contributed not a little to his professional success. In the month of September, 1834, the writer of this article was walking, one fine day, down the principal street of Berne, in Switzerland, when he observed a fine tall and majestic figure slowly pacing the middle of the same street, while numbers of people on both sides were constantly stopping to look at this object of general attention. Even the ladies were throwing open the sashes of their windows, and admiring the noble-looking stranger, who in his turn was contemplating, with much curiosity, and apparent enjoyment, the singular and picturesque costume of the Bernese. By a side-glance, the writer recognized his old friend and preceptor, and stealing up quietly behind, gave the Baronet a very smart slap on the shoulder, to the no small amusement of the Bernese loungers and spectators. A blowse-frock, travelling cap, and bronzed complexion, so masked the old friend, that Sir Astley was utterly astonished at the liberty which a stranger had taken in the middle of the street. Matters, however, were easily explained, and the writer strongly urged the Baronet to make excursions among the mountains with him, in search of health, rather than plod among museums and hospitals, of which he must have had enough and to spare. But no. Sir Astley had rather breathe the mephitic atmosphere of the dead-house or dissecting-room, than inhale the invigorating breeze of alpine height, or glittering glacier. It is probable that had this highly talented surgeon spent a couple of months annually in travelling exercise, he would have greatly checked the ravages of gout, and one of its most fatal sequences—disease of the heart.

It is quite useless to advert to Sir Astley Cooper's writings or lectures. They are well known, and need no eulogy or even notice. Many of the gossipping facts that have been stated in print, since Sir Astley's decease, are mere *fictions*. The anecdote of his demanding a shilling from an old patient who had tendered him a sovereign, is, we have no doubt, totally devoid of foundation. Till the very last illness, Sir Astley was eager after practice, and scarcely ever declined it, even when the case was purely medical; but it was not the thirst for fees—it was the love of avocation that led him to die with harness on his back. It is quite evident that the early habits and active life of this eminent practitioner disqualified him for literary leisure in advanced age. Even the salutary and amusing exercise of travelling, which his fortune enabled him to pursue, was not an equivalent for the professional avocation which had become a part and parcel of this gifted individual's constitution.

Sir Astley Cooper was one of those (not too numerous) who always acted with liberality and honesty towards his brethren, whether behind their backs or before their faces. The default in this Christian and humane conduct has injured, and continues to injure the medical character more than all the abuses which have crept into our institutions, and all the other imperfections that pervade our ranks! This amiable trait in Sir Astley's character will long render his memory cherished by a wide circle of his professional brethren, and will cause even more regret than the loss of his professional skill and acquirements to the community. Sir Astley was the last King Chirurgical which England will ever see. The advances and the diffusion of general, as well as medico-chirurgical education, have now worked a greater equilibrium of knowledge among the profession than ever before existed. We may, and, no doubt, always will have an *aristocracy* of talent and reputation; but never an *autocracy* again! It will be the same with professional income. We much doubt whether any surgeon in this metropolis will ever receive *half* the annual income which Sir Astley Cooper received for many years in succession. For his princely fortune he was *partly* indebted to the times in which he lived—but *chiefly* to natural talent, inextinguishable zeal, unwearied industry, liberality of conduct to his brethren, and courteous demeanor to all. These are the honorable characteristics that ought to be engraven on his tomb!

---

Egypt and Mohammed Ali, illustrative of the Condition of his Slaves and Subjects, &c. By R. R. Madden, M.D. Octavo, 1841. Hamilton and Co.

Our old friend, Dr. Madden, whose exertions in the cause of humanity were so successful in the West Indies, is now on his way to the Niger, with the exploratory expedition to that land of sickness and slavery. As an interlude between Cuba and Cape Coast Castle, our highly talented and adventurous friend accompanied Sir Moses Montefiore on his mission to Damascus, with the view of rescuing the Jews of that once famous city from the iniquitous persecution of their Mohammedan tyrants—a persecution, we grieve to say, instigated, prompted, witnessed, and approved by the representative of *liberal* France —Mons. Menton, the Consul General of the "Grande Nation!!" This worthy employée of the candid and ingenuous Thiers managed to see a few of the poor Israelites scourged to death, or *tortured,* till they confessed that of which they were no more guilty than was the Queen of England! Such are the enlightened views of jeune France! But as this subject will be better handled by the lay press than by us, we shall say no more on it.

Dr. Madden was once more at home in the East, after an interval of many years, and his little volume will be read with great interest by all who have at heart the liberation of our fellow-men from the chains of slavery, whether in Egypt, Africa, or the Antilles. There is but one short chapter in the work which bears directly on medicine—and that is, "On the Plague at Alexandria in 1840."

Dr. Madden met with his old acquaintance, Dr. Grassi, after an interval of fifteen years, in the plague hospital, where he had officiated during that long period. There are, perhaps, few medical men now living, who have had such ample opportunities for observation on this interesting subject, as Dr. Grassi. "He is a plain practical man, of strong sound sense, and one whose opinion on the subject of contagion or non-contagion, deserves more attention than that of any man alive."

"Dr. Grassi believes that the plague is contagious, and it seems to me impossible for any man in his senses to hear Grassi's opinions given on this subject—opinions founded on experience alone, and the strong arguments and facts which he brings forward in support of those opinions, and not conceive with him, that this disease is propagated from one person to another, and that the separation of the sound from the sick, is requisite to prevent the disease from spreading through the community. Clot Bey has written, recently, a large work on this subject, in which he controverts these opinions, but he has proved nothing, and his opinions are not those of a practical observer of this disease."

Dr. Grassi and Dr. Madden agree that the injuries done to commerce by the present quarantine are great, and, in a great measure, unnecessary. It is the opinion of Dr. Grassi and of all the contagionists in Egypt, that the miasma is quite innoxious after ten days, whether in clothes or goods. Not one of them ever knew the disease to be propagated by infection after 20 days from the time of contact. The head of the quarantine department at Malta assured Dr. Madden that he never saw a single case of plague being communicated in the Lazaretto there, under any circumstance whatever. The same authority thinks that eight or ten days' quarantine are amply sufficient for the protection of the island against plague. On this side of Gibraltar no quarantine at all is necessary, where there are clean bills of health.

A TABLE
Exhibiting the Progress and Decrease of the Plague.

| | |
|---|---|
| January, received .. .. .. .. .. .. .. .. .. .. | 5 |
| February ,, .. .. .. .. .. .. .. .. .. .. | 15 |
| March ,, .. .. .. .. .. .. .. .. .. .. | 57 |
| April ,, .. .. .. .. .. .. .. .. .. .. | 179 |
| May ,, .. .. .. .. .. .. .. .. .. .. | 162 |
| June ,, .. .. .. .. .. .. .. .. .. .. | 48 |
| July ,, .. .. .. .. .. .. .. .. .. .. | 27 |
| August ,, .. .. .. .. .. .. .. .. .. .. | 2 |
| In the Plague Hospital of the Lazaretto .. .. .. .. .. Total | 495 |

In the City, the Port, and the Suburbs of Alexandria, about 1000 cases more.

Should our amiable and highly accomplished author ever return from the Niger expedition, (which we fervently hope he will,) there is little doubt that we shall have a volume of highly interesting information from that "undiscovered bourne," from whence, alas! but few return to tell the tale!

---

The Domestic Management of the Sick-room, necessary in aid of Medical Treatment, for the Cure of Diseases. By A. T. Thomson, M.D. &c. &c. Octavo, pp. 506. Longman & Co. 1841.

This volume is dedicated to the "Ladies of Great Britain," and "intended to render efficient their duties in the sick-room." To the said ladies it is one of the most valuable gifts that have ever been presented from the pen of a talented, old, and experienced practitioner. But the ladies of Great Britain are not the only class who ought to study this volume. The whole of the young practitioners—and nine-tenths of the old ones—will here find an immense magazine of "useful knowledge," which will often prove more serviceable and available than the armamenta medicaminum of the Pharmacopœia. The prescriptions of the physician are very often rendered nugatory, if not injurious, by the ignorance of the nurses and friends of the sick—and it is equally the duty and the interest of the medical practitioner to peruse and recommend this volume, as a work that will conduce to the recovery of his patient and the credit of himself. Unlike the generality of popular medical works, this of Dr. Thomson's will tend to smooth the path of the medical attendant by edifying and instructing the nurses and friends of the sick. We shall probably extract some passages in a future number, as samples of the work; but the name of the author, and the intrinsic merits of the performance, will insure it an extensive circulation.

